# The Calcium-Dependent Protein Kinase 3 of *Toxoplasma* Influences Basal Calcium Levels and Functions beyond Egress as Revealed by Quantitative Phosphoproteome Analysis

**DOI:** 10.1371/journal.ppat.1004197

**Published:** 2014-06-19

**Authors:** Moritz Treeck, John L. Sanders, Rajshekhar Y. Gaji, Kacie A. LaFavers, Matthew A. Child, Gustavo Arrizabalaga, Joshua E. Elias, John C. Boothroyd

**Affiliations:** 1 Department of Microbiology and Immunology, Stanford University School of Medicine, Stanford, California, United States of America; 2 Department of Chemical and Systems Biology, Stanford University School of Medicine, Stanford, California, United States of America; 3 Department of Pharmacology and Toxicology, School of Medicine, University of Indianapolis, Indianapolis, Indiana, United States of America; 4 Department of Pathology, Stanford University School of Medicine, Stanford, California, United States of America; University of Geneva, Switzerland

## Abstract

Calcium-dependent protein kinases (CDPKs) are conserved in plants and apicomplexan parasites. In *Toxoplasma gondii*, TgCDPK3 regulates parasite egress from the host cell in the presence of a calcium-ionophore. The targets and the pathways that the kinase controls, however, are not known. To identify pathways regulated by TgCDPK3, we measured relative phosphorylation site usage in wild type and TgCDPK3 mutant and knock-out parasites by quantitative mass-spectrometry using stable isotope-labeling with amino acids in cell culture (SILAC). This revealed known and novel phosphorylation events on proteins predicted to play a role in host-cell egress, but also a novel function of TgCDPK3 as an upstream regulator of other calcium-dependent signaling pathways, as we also identified proteins that are differentially phosphorylated prior to egress, including proteins important for ion-homeostasis and metabolism. This observation is supported by the observation that basal calcium levels are increased in parasites where TgCDPK3 has been inactivated. Most of the differential phosphorylation observed in CDPK3 mutants is rescued by complementation of the mutants with a wild type copy of TgCDPK3. Lastly, the TgCDPK3 mutants showed hyperphosphorylation of two targets of a related calcium-dependent kinase (TgCDPK1), as well as TgCDPK1 itself, indicating that this latter kinase appears to play a role downstream of TgCDPK3 function. Overexpression of TgCDPK1 partially rescues the egress phenotype of the TgCDPK3 mutants, reinforcing this conclusion. These results show that TgCDPK3 plays a pivotal role in regulating tachyzoite functions including, but not limited to, egress.

## Author Summary

Calcium-dependent protein kinases are plant-like enzymes of apicomplexan parasites that regulate a variety of biological processes including stage-conversion, post-translational repression and egress from the host cell. In this study, we analyzed *Toxoplasma* CDPK3, which has recently been shown to regulate rapid egress from the host cell. The specific pathways that TgCDPK3 regulates, however, have not previously been known and so we used a quantitative phosphoproteome approach to determine phosphorylation site usage in wild type and TgCDPK3 mutant parasites before, during and after egress. This revealed >150 novel phosphorylation sites that are differentially phosphorylated between WT and TgCDPK3 mutant parasites. Some of these sites are on proteins predicted to play a role in parasite egress. However, we also identified many phosphorylation sites on proteins not thought to be involved in egress, as well as many proteins of unknown function. We confirm that basal calcium levels are affected by CDPK3 inactivation and observed a link between TgCDPK3 and another calcium-dependent kinase (TgCDPK1). Known targets of TgCDPK1 were hyperphosphorylated in the TgCDPK3 mutants, and overexpression of TgCDPK1 partially rescued the observed egress phenotype of TgCDPK3 mutants.

## Introduction

Apicomplexan parasites like *Toxoplasma gondii* and *Plasmodium* species contain a number of plant-like calcium-dependent protein kinases (CDPKs) [1]. These have been shown to be druggable targets that are distinct from their mammalian hosts. For that reason and for their suggested role in regulating calcium-dependent processes in apicomplexan parasites, CDPKs have been the object of intense study. For example, several reports have used either direct, conditional or chemical knock-out strategies to investigate the function of CDPKs in *T. gondii* and *Plasmodium* [2]–[11].

In *T. gondii*, two CDPKs have been investigated in detail. TgCDPK1 (TGGT1_059880) has been shown to be essential for the secretion of micronemes [5] and in three independent studies, TgCDPK3 (TGGT1_041610) has recently been shown to be important for regulating the rapid egress from the host cell upon treatment with the calcium ionophore A23187 [6]–[8], [12]. TgCDPK3 mutants show retarded microneme-secretion and ionophore-induced egress although they still extrude the conoid, a complex structure in the apical part of the parasites, with the usual kinetics [13] [6], [7]. These results show that the mutant parasites can sense the calcium signal induced by the ionophore to some extent but fail to transduce that signal in the normal way. The fact that egress does still occur in the TgCDPK3 mutants, albeit with a delay, indicates that other signaling pathways can operate in the absence of this enzyme [6].

A second phenotype that is associated with TgCDPK3 malfunction is resistance to ionophore-induced death (IID) [13]. IID describes the sensitivity of extracellular tachyzoites to prolonged treatment with a calcium-ionophore. All parasite lines identified harboring mutations in the *TgCDPK3* gene show resistance to IID, but the biochemical basis for this is not yet known. TgCDPK3 also appears to be necessary for producing latent stages in mice [14], but the mechanism is also not yet understood.

To understand the role of TgCDPK3 during both normal conditions and ionophore-induced egress, we performed a quantitative phosphoproteome and proteome study of wild type and TgCDPK3 mutants using stable isotope labeling with amino acids in cell culture (SILAC). Our study revealed clues to the role played by this enzyme in normal physiology and induced egress.

## Results

### SILAC-based quantitative phosphoproteome and proteome analysis

**Figure 1:**
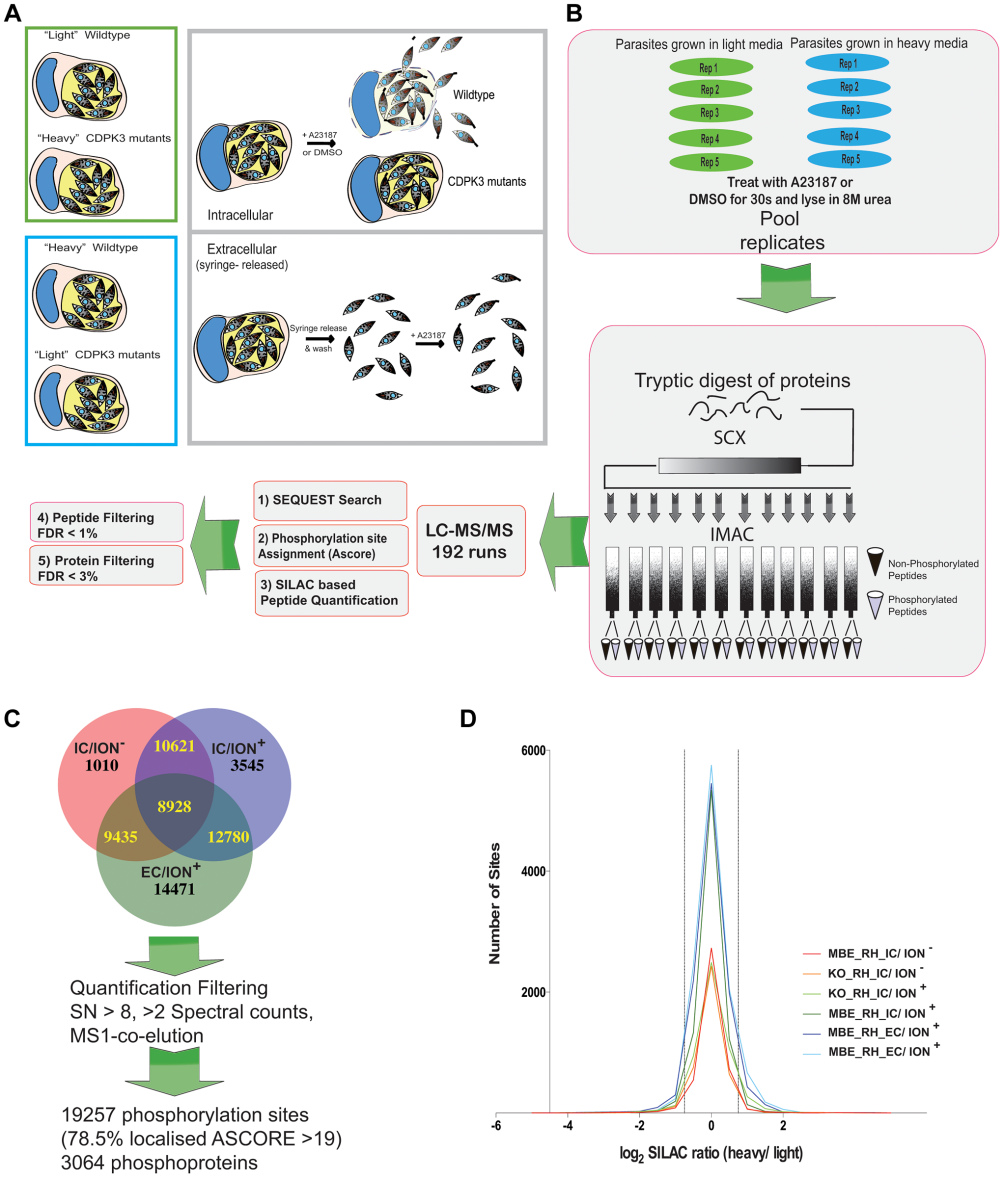
Generation of phosphoproteome samples. **A**) Illustration of the experimental conditions used in this study and the functional consequences of treatment with the calcium ionophore A23187. Upper panels show “intracellular” conditions: infected cells containing mutant or wild type parasites were labeled with “heavy” or “light” SILAC media, treated with A23187 ionophore or DMSO for 30 sec and subsequently lysed for phosphoproteome analysis. Whereas wildtype parasites rapidly egress upon ionophore treatment (after 2 minutes, 100% have exited the host cell), TgCDPK3 mutant parasites extrude the conoid, a sign that they have sensed the ionophore, but fail to egress in a timely matter. Lower panels show “extracellular” conditions: all as for “intracellular” except the cultures were scraped and passed through a syringe to release the parasites followed by ionophore treatment for 30 seconds. All conditions were performed in “forward” and “reverse” (where the heavy and light labeling are swapped). **B**) 5 dishes (“rep”) per condition (or 5 vials containing extracellular parasites) were individually treated for 30 seconds prior to lysis, mixing, reduction and alkylation. SCX chromatography and IMAC were used for phosphopeptide enrichment. Blue and green color-coding represents parasites grown in “light” or “heavy” media, respectively. In total, 96 samples were analyzed in duplicate by LC-MS/MS followed by bioinformatic filtering to reduce the site and protein FDR to <1% and 3%, respectively. **C**) A total of 32,147 phosphorylation sites were identified in the three datasets: intracellular treated with ionophore (IC/ION^+^) or DMSO (IC/ION^−^) and extracellular treated with ionophore (EC/ION^+^). Further filtering based on criteria specified in the text, including signal/noise ratio >8 (SN>8) reduced the set of quantified phosphorylation sites to 19,257 in 3064 proteins (See also Table S1). **D**) Histogram of median-centered log_2_ H/L SILAC ratios for each dataset comparing a TgCDPK3 mutant and wild type RH. MBE indicates the MBE1.1 mutant that is resistant to the ionophore and KO indicates the TgCDPK3 knock-out mutant. The conditions are as described above.

To identify the pathways controlled by TgCDPK3, we infected human foreskin fibroblasts (HFFs) with wild type (WT) or TgCDPK3-mutant tachyzoites previously grown in either “heavy” (H) or “light” (L) conditions. “Heavy” indicates the presence of ^13^C,^15^N-Lys in place of the naturally-occurring (^12^C or ^14^N) amino acids in “light” media [15], [16]). Approximately 24 h after continued growth in either “heavy” or “light” media, the infected cells were incubated for 30 seconds in the presence of 1 µM calcium-ionophore or DMSO as a control (Figure 1A). These samples are called “intracellular”. We also compared WT and mutant parasites grown under “heavy” or “light” conditions and then exposed to ionophore after being released from the host cells by syringe lysis. These samples, called “extracellular,” were used to examine the role of TgCDPK3 in ionophore-induced death and gliding motility, a process by which the parasites use their own motor-proteins to glide over a surface.

In total, we generated 6 datasets (experiments 1–6, see Supplemental Figure S1 and Supplemental Table S1). These compared WT and mutant parasites under each of three conditions in technical duplicate: 1) intracellular parasites without ionophore (“IC/ION^−^”); 2) intracellular parasites with ionophore (“IC/ION^+^”); and 3) extracellular parasites with ionophore (“EC/ION^+^”). We measured each condition with forward and reverse labeling; that is, we labeled WT parasites with “light” amino acids and the TgCDPK3-mutant parasites with “heavy” in one experiment and reversed this labeling for the replicate experiment (Figure 1A and Supplemental Figure S1). This strategy ensured that host-cell peptides, which are abundantly present in the “intracellular” samples, did not introduce quantification errors; reverse labeling effectively removes false positives resulting from misidentified host-derived peptides, since they will show the same heavy/light ratio, irrespective of the labeling. True, parasite-derived quantifications will have reciprocal log_2_ H/L ratios for the reverse-labeled experiment.

Knock-out mutants of TgCDPK3 (RH:*Δcdpk3* [8]), but not the point mutants resulting from chemical mutagenesis (e.g., MBE1.1 [13]), show impaired growth relative to WT parasites. This phenotypic difference led us to use *Δcdpk3* and MBE1.1 in different experiments (see Supplemental Figure S1) in our study as phosphorylation sites that differ between WT and both *Δcdpk3* and MBE1.1 are unlikely to be caused by either the growth defect observed for *Δcdpk3* or a mutation in the chemical mutants outside of the *TgCDPK3* gene. To ensure that any change in phosphorylation site abundance is not simply a result of a change in the general abundance of that protein, we also measured non-phosphorylated peptides (for the remainder of the manuscript called the “proteome”) from two experiments.

We analyzed 192 LC-MS/MS runs from 6 different experiments under three different conditions to identify signaling pathways that are differentially regulated between WT and TgCDPK3 mutant parasites: 72 phosphoproteome samples, and 24 proteome samples from two experiments (“IC/ION^−^” and “EC/ION^+^”), all analyzed in two independent runs (i.e., in “technical duplicate”; Supplemental Table S1).

We identified differences in protein levels and phosphorylation site usage between WT and TgCDPK3 mutant strains using a phosphopeptide-enrichment strategy [17] which we previously applied to *Toxoplasma* parasites [18]. All datasets were initially filtered to a false discovery rate (FDR) of <1% on the peptide and <3% on the protein level. In total, we identified 32,147 phosphorylation sites (Figure 1C), 69.4% of which we previously identified in phosphoproteomic studies of WT tachyzoites [18]. Primary analysis of the data obtained revealed up to 50% FDR of “decoy” hits (obtained by searching a fictional decoy database consisting of reversed protein sequences [19]) in phosphorylation sites with a very high or low H/L SILAC ratio even though the total set FDR was well below 1% across all datasets. To largely eliminate false positives from these “tails” (high or low SILAC ratios), we further filtered all quantifications for the signal to noise ratio, spectral counts and MS1-elution parameters of the SILAC pairs (see materials and methods). After such filtering, 19,257 sites remained which were considered quantified with a site FDR of 0.41% and a protein FDR of 1.61% (see Supplemental Table S1). Note that the FDR refers to the estimated number of incorrect identifications of phosphopeptides in the dataset as a whole and does not estimate the correctness of all quantifications, so additional filtering, as described above, is required for highly reliable quantifications. We include all quantifications after the above-mentioned filtering in this manuscript (Table S1) because these represent a resource that can inform the interpretation of complementary approaches to identify targets of TgCDPK3. 78.5% of all quantified sites identified have an ASCORE >19, indicating a 99% probability of being correctly localized to an S, T or Y residue within the peptide they were found [20]. The median-centered SILAC ratios of all quantified sites show a normal Gaussian distribution with a median standard deviation of ∼0.5 for each experiment (Figure 1D), showing that the presence of mostly unlabeled host-cell material in our samples did not skew the expected distribution of SILAC ratios at a detectable level.

We identified a subset of phosphorylation sites as reliably different between WT and mutant parasites by applying two additional criteria: 1) the site was identified in at least two datasets without conflicting ratios for different experiments (e.g., they must have a positive log_2_ H/L ratio in a forward and a negative log_2_ H/L ratio in the reverse experiment); 2) the log_2_ H/L ratio was >0.75 or <−0.75, (∼1.5 times the average standard deviation of any given experiment); sites were designated as “not different” if the log_2_ ratios were between 0.5 and −0.5. Sites were considered dependent on TgCDPK3 if they differed between WT and mutant parasites under one or more of the three experimental conditions, “IC/ION^−^”, “IC/ION^+^” or “EC/ION^+^”.

Given that treatment with ionophore was for 30 s followed by a wash, minor differences in the timing of the wash could contribute to considerable phosphorylation state variation between datasets. Thus, we allowed one unexpected value in any of the conditions. Using this filtering, we obtained a list of 156 phosphorylation sites that are different between WT parasites and TgCDPK3 mutants with a final FDR of 0.5% (Supplementary Table S2).

### TgCDPK3 has a regulatory function in parasite physiology in addition to ionophore induced egress

**Figure 2:**
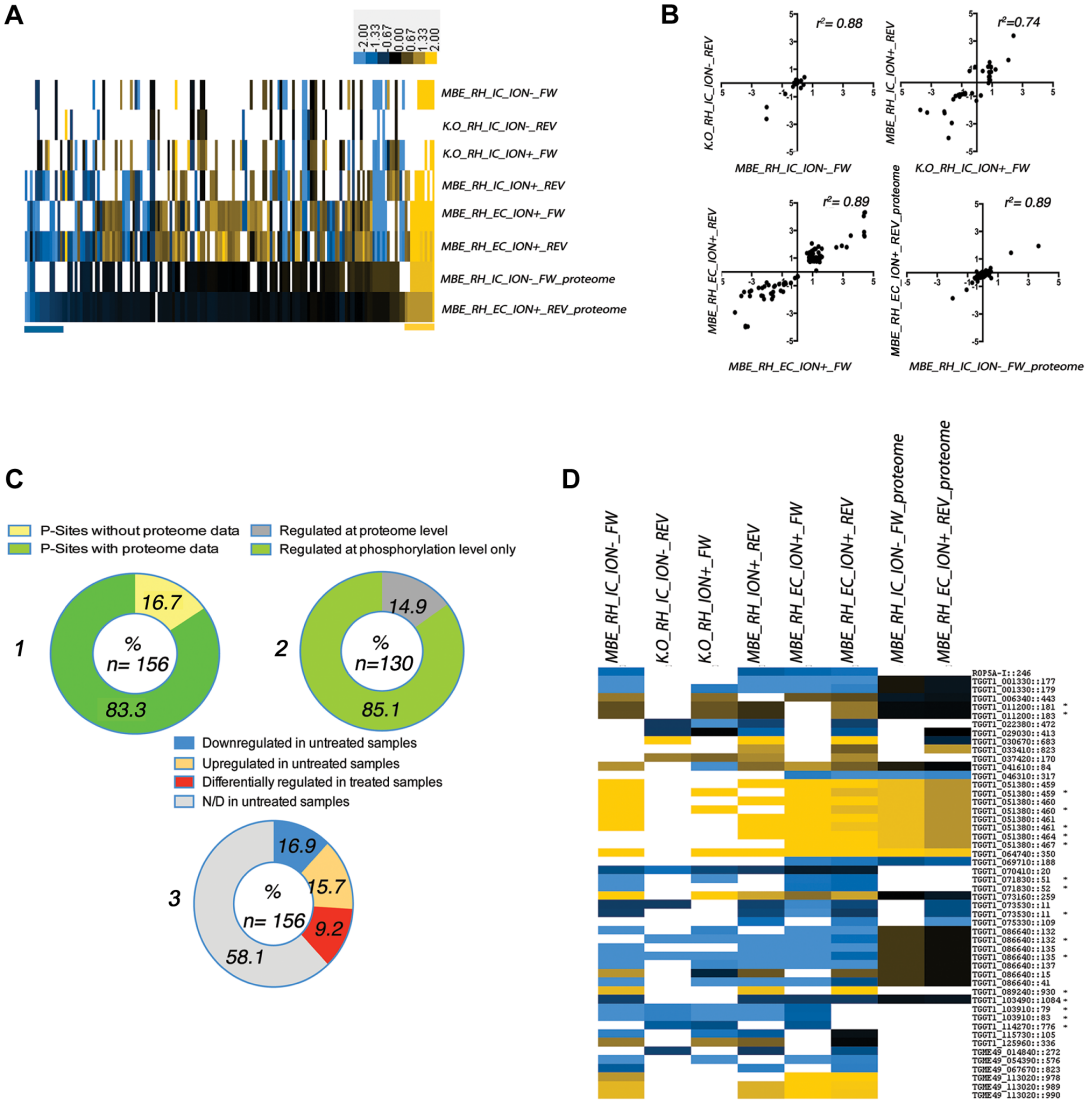
Analysis of differential phosphorylation site usage and protein abundance in WT and TgCDPK3 mutant parasites (see **Figure 1** and Figure S1 for a description of experimental conditions). **A**) 156 phosphorylation sites were identified as different between WT (RH) and TgCDPK3 mutant parasites under one or more of the conditions described in Fig. 1. FW = forward; REV = reverse labeling. The heat-map represents the log_2_ H/L SILAC ratios for the phosphoproteome or, where indicated, the proteome experiments. Each bar represents a phosphorylation site. Missing values are white. Only phosphorylation sites for which protein levels were also measured are displayed. A positive log_2_ score indicates higher abundance of the phosphopeptide in mutant compared to WT (RH) parasites. The results are sorted first for increasing SILAC ratios on the protein level (see also Table S2). Bars at the bottom of the heat map indicate phosphorylation sites where the difference in phosphorylation state can be explained by differences on the protein level. The color bar represents log_2_ fold-changes. **B**) Pearson correlation of phosphorylation sites and protein SILAC-ratios between WT (RH) and TgCDPK3 mutants identified in each condition (forward and reverse for “IC/ION^−^”, “IC/ION^+^” and “EC/ION^+^”). Each dot represents one phosphosite and the log_2_ ratio under any one condition but with forward labeling being one mutant relative to WT and the reverse labeling being the other mutant relative to WT. **C**) 1) Percentage of the 156 differing phosphosites for which proteome information was also obtained; 2) Percentage of the 130 differing phosphosites for which proteome information was also obtained where the differences in the protein's abundance was (“Regulated at proteome level”) or was not (“Regulated at phosphorylation level only”) sufficient to explain the difference in the abundance of the phosphorylated peptide; 3) Distribution of phosphorylation sites regulated in the absence or presence of ionophore. Blue and yellow indicate the percentage of sites that appear less or more phosphorylated in the mutant samples relative to wild type in the “IC/ION^−^“ conditions (“untreated”). Red indicates the percentage of sites that were identified as differentially phosphorylated in the “IC/ION^+^” conditions, but phosphorylated at similar levels between WT and mutant parasites in the “IC/ION^−^“ conditions. Grey indicates phosphopeptides that were not detected (N/D) in the untreated samples. **D**) Heat-map showing all phosphorylation sites and proteins with changing phosphorylation ratios in “IC/ION^−^”conditions, sorted by annotated gene identifier based on ToxoDB (v7.3). Samples are annotated as in Fig. 2A. The numbers next to the annotation indicate the position of the phosphorylation site within the protein. An asterisk (*) following the gene ID indicates quantifications are based on the bis-phosphorylated peptide form. The heat-map color intensities correspond to Figure 2A.

The criteria above yielded 156 phosphosites with abundances that differed between wild type and mutant parasites under any of the three conditions; for 130 of these we also obtained protein-level data (i.e., SILAC ratios for nonphosphorylated peptides from the same protein; Figure 2A ad 2C1 and Table S2). Pearson-correlation analysis of the forward and reverse experiment for each condition showed significant correlation (p-values <0.0001 for all comparisons) (Figure 2B). These results allowed us to identify which differences in phosphopeptide abundance can be explained simply by a difference in the abundance of that protein (Figure 2C); only ∼14.9% of the 130 phosphosites where we also had proteome data correlated with protein abundance, showing that the vast majority of sites identified as different in the mutants are likely a result of differences in the degree of phosphorylation (Figure 2C2 and Supplemental Figure S2).

For a majority (∼58%) of the 156 phosphorylation sites discussed above we did not obtain high-confidence SILAC ratios for the untreated samples (“IC/ION^−^”; Figure 2A and 2C3), precluding any conclusion about their regulation in the absence of ionophore. In the 65 phosphosites where we did obtain such data, however, we observed the following: only 2 of the proteins on which one or more of these phosphosites were detected showed a difference in protein levels and both these were in the set that was different in the “IC/ION^−^” conditions. 51 of the 65 phosphosites showed a significant difference between wild type and mutant parasites even in the absence of ionophore (Figure 2C3) and only 14 were not significantly different in these latter conditions. These results indicate that TgCDPK3 likely regulates biological processes during the normal function of intracellular parasites, independent of egress and ionophore treatment. Among the phosphorylation sites that are already different in the absence of ionophore are some on proteins important for ion-homeostasis (P-type ATPase, putative, TGGT1_103910) and a dense granule protein GRA22 (TGGT1_125960) that has recently been shown to play a role in egress [21]. Importantly, many of the differences were observed in the two independently derived TgCDPK3 mutant lines (MBE1.1 and RH*Δcdpk3*) indicating that these changes are a consequence of TgCDPK3 inactivation and not a consequence of the genetic modification of the parasites independent of TgCDPK3 function (Figure 2D).

### Complementation of MBE1.1 with wild type TgCDPK3 largely restores differential phosphorylation but not changes in protein abundance

The two datasets for “EC/ION^+^” contained most of the 156 differing phosphorylation sites, but these experiments involved comparisons between RH (wild type) and the same mutant strain (MBE1.1). Thus, we could not exclude the possibility that some of the observed differences in phosphorylation state are due to secondary mutations carried by this strain (i.e., are not dependent on TgCDPK3). To address this, we made use of a complemented MBE1.1 cell line which expresses wild type TgCDPK3 under its endogenous promoter [8] and compared the phosphoproteome and proteome of WT and MBE1.1::CDPK3 from extracellular, ionophore-treated parasites in a new experiment “COMP/EC/ION^+^” using the methods described above. We then retrieved all SILAC ratios of this experiment for the 156 phosphorylation sites that we identified as different between WT and CDPK3 mutant parasites (Supplemental Table S2).

**Figure 3:**
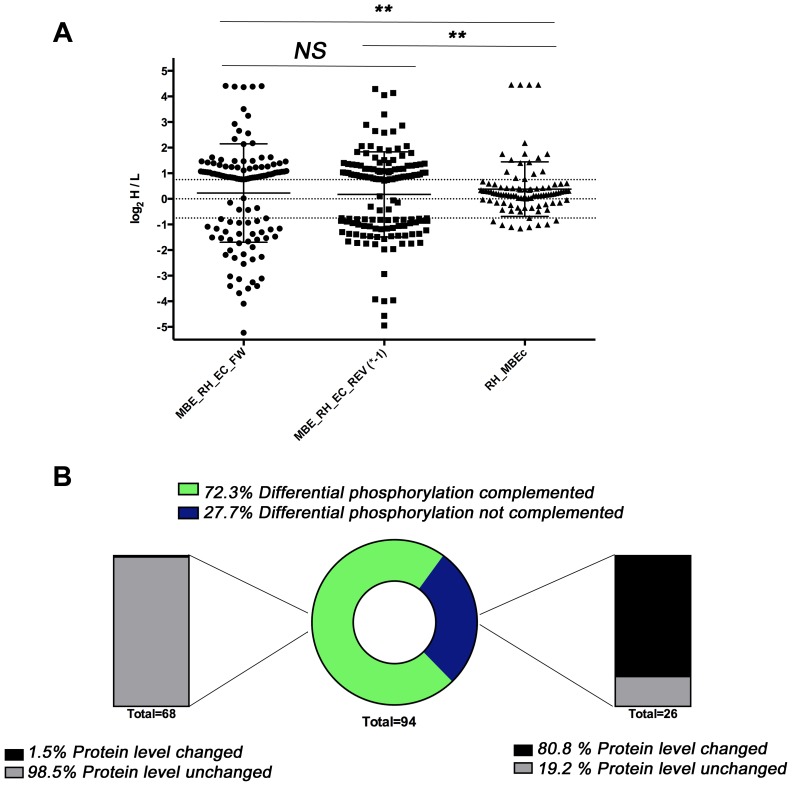
Phosphoproteome and proteome analysis of MBE1.1 complemented with wild type TgCDPK3. **A**) All log_2_ H/L SILAC ratios for the 156 phosphorylation sites identified as differentially phosphorylated between TgCDPK3 mutants and wild type parasites are plotted from both extracellular phosphoproteome experiments and the corresponding SILAC ratios from the comparison of WT (RH) and the MBE1.1::CDPK3 complemented mutant (MBEc). Each point represents an individual phosphosite. The distribution of the SILAC ratios was not significantly different (NS) between the forward and the reverse experiments where MBE1.1 and RH parasites were compared. The RH vs. MBEc comparison showed most phosphosites were not significantly different between these two samples (log_2_ H/L values between 0.75 and −0.75) and the overall dataset was significantly different relative to the two comparing RH and the MBE1.1 mutant (**; p<0.001). **B**) Pie/bar charts showing that the majority of phosphorylation sites are complemented. Of the 156 TgCDPK3-dependent sites described in Fig. 2A, 94 gave reliable data in the comparison between the complemented mutant and WT strains. The central pie chart shows the percentage of these that were rescued by complementation (i.e., were no longer different relative to RH (WT); black) or were not rescued (i.e., still differed relative to WT; grey). The left bar shows that of the 68 (72.3%) sites that were complemented, only 1 (1.5%) was also changed in regards to the overall protein abundance. In contrast, a majority (20 or 80.1%) of the 26 phosphosites that were not rescued by complementation differed in protein abundance between WT and mutant (right bar).

As expected, we observed a significant difference (P-value <0.0011 Kolmogorov-Smirnov test) between the distributions of SILAC ratios of all 156 differing phosphorylation sites of the “EC/ION^+^” datasets for the mutant (MBE_RH_EC_ION^+^_forward or reverse) when compared to the SILAC ratios observed for “COMP/EC/ION^+^” (Figure 3A). Overall, 68 (72.3%) of the 94 phosphorylation sites for which we obtained SILAC ratios from the complementation experiments showed complementation (Figure 3B). Of the 26 sites that were not rescued in the complemented strain, the vast majority (80.8%) appeared different because of differences in protein-levels compared to only 1.5% of the sites that were complemented. These data show that differences in the efficiency of phosphorylation at a given site in the TgCDPK3 mutant are largely complemented and differences that occur at the protein-level are not. While these latter differences in protein abundance could be an indicator of MBE1.1-specific effects due to undetected mutations or passage history, we observed that ∼25% of the sites that were not complemented were also identified as differentially phosphorylated in RH*Δcdpk3* vs. WT parasites, indicating a dependence of TgCDPK3 function.

### Ionophore treatment reveals phosphorylation sites potentially involved in the onset of egress

**Figure 4:**
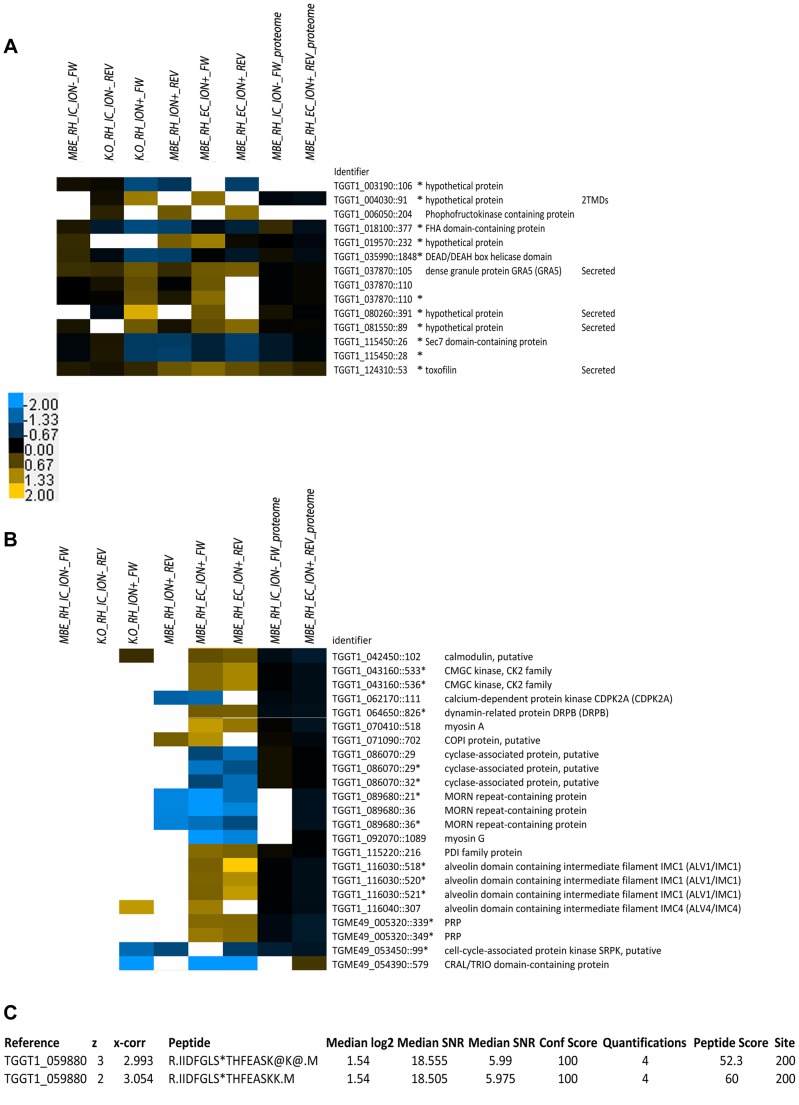
Heat-map of phosphorylation sites regulated during egress. **A**) Comparison of phosphorylation sites that show an ionophore-dependent difference between WT vs. mutant TgCDPK3 only when treated with ionophore, but equal phosphorylation site usage in the absence of ionophore. Conditions are as for Fig. 1 and 2. The color bars represent log_2_ H/L fold-changes for each. Identifier indicates the gene annotation and location of the site within the protein. An asterisk indicates whether a phosphorylation site has been identified on a bis-phosphorylated peptide. **B**) Heat-map of phosphorylation sites suspected or known to be involved in egress or motility. Details as for Part A except the phosphosites shown are all those that are not identified in the absence of ionophore and different when treated and were identified on proteins implicated in egress or motility. **C**) Phosphopeptide isoforms identified and quantified in this study containing the autophosphorylation (T200) from TgCDPK1. Median values for all peptide measurements are shown. The phosphorylated residue is marked (*).

A primary aim of this study was to identify phosphorylation sites that differ in their usage between the WT and TgCDPK3 mutant parasites upon ionophore treatment. Within this set of 156 changing sites, two classes are most interesting: “Class A” where the phosphosite was detected in both WT and mutant parasites in the absence of ionophore-treatment but whose usage did not differ between these two strains; and “Class B” where the phosphosite was not detected in one or other or both of the strains in the absence of ionophore-treatment. This latter class likely represents phosphorylation sites that are substantially phosphorylated only during ionophore-induced egress, assuming the absence of detection in the “IC/ION-“ condition is not caused by technical reasons, as explained below. In Class A, we identified 14 phosphorylation sites on a total of 11 proteins (Figure 4A). Seven of the 11 proteins are more phosphorylated in the mutants relative to WT in the presence of ionophore and 5 of these were predicted or shown to be secreted into the host cell [18]. The preponderance of secreted proteins in this group is discussed further below.

To understand more about TgCDPK3's role, we looked for proteins in our dataset that were already known or predicted to play a role in egress or motility (Figure 4B). Among the identified proteins in Class B are several that are associated with actin regulation (cyclase associated protein (CAP, TGGT1_086070) [22]–[24]), putative motor-proteins (Myosin A (TGGT1_070410), F (TGGT1_103490) and G (TGGT1_092070) [25]), proteins of the inner membrane complex (IMC [26]) and a recently discovered protein that associates with cortical microtubules (TrxL-1 (TGGT1_115220) [27]). Although IMC and microtubule-associated proteins are not directly implicated in egress or motility, rapid rearrangements of the cytoskeleton prior to egress could be part of such a process. Also two uncharacterized kinases (TGGT1_043160, TGME49_053450) were identified, although nothing about their function is known.

Whether TgCDPK3 inactivation leads to a motility phenotype in the presence of ionophore remains unclear. Whereas Lourido and colleagues reported significant differences in the types of motility that TgCDPK3 mutants could perform, McCoy and colleagues observed only a slight, but not significant trend toward such differences [6], [7]. Both groups reported no differences in the speed of the parasites while gliding over a surface. Several members of the motor-complex (MyoA, GAP45 (TGGT1_078320) and MLC1 (TGGT1_013010)) are known to be phosphorylated in a calcium-dependent manner in *Toxoplasma* and *Plasmodium* [28]–[33]. Thus, we specifically looked for differences in the relative abundance of phosphopeptides in such proteins as well as other members of the motor complex: GAP40, GAP50, GAP70 and aldolase [34], [35]. Many previously identified phosphosites in these proteins were identified in our datasets but only MyoA and Aldolase (TGGT1_069710) showed a difference in relative phosphorylation between the TgCDPK3 mutants and WT parasites: on MyoA we observed increased phosphorylation of S518 and decreased phosphorylation of S20/S21 in extracellular, ionophore-treated mutants vs. WT (we cannot differentiate which of the two adjacent serines is phosphorylated based on the spectra). The latter site was less phosphorylated in the mutants in the absence of ionophore but this difference decreased in the “EC/ION^+^” condition. These data suggest that while TgCDPK3 activity is essential for processes that regulate egress-related events, it is not the main kinase regulating phosphorylation of the motor complex components.

### Inactivation of TgCDPK3 causes differential phosphorylation in calcium-controlled proteins and indicates an activating function of TgCDPK3 in the pathway controlled by TgCDPK1

Among the proteins that were differentially phosphorylated between wild type and mutant parasites were some that contain EF-hands, proteins that are regulated directly by calcium (Figure 4B). We identified a differentially regulated phosphorylation site on a small EF-Hand protein that contains no other recognizable domain and is annotated as a putative calmodulin (TGGT1_042450). The phosphorylation site was identified just adjacent to the EF-hands themselves.

In addition to the putative calmodulin, we identified two calcium-dependent kinases (TgCDPK2a (TGGT1_062170) and TgCDPK3 itself) as differentially phosphorylated in the MBE1.1 mutant vs. WT. For both kinases, the sites were hyperphosphorylated in the mutant parasites and were located within the ATP-binding loop of the kinase domain, a region where phosphorylation can play a regulatory role [36], [37]. It is worth noting that the mutation (T239I) in the activation loop of TgCDPK3 in the mutant MBE1.1 [8] functionally inactivates the kinase and so a different kinase must be involved in the hyperphosphorylation.

Interestingly, our dataset also indicated that two of several phosphorylation sites on proteins recently identified as targets of TgCDPK1 (TGGT1_059880). PRP (TGME49_005320) and DRPB (TGGT1_064650) [38] are, surprisingly, more phosphorylated in TgCDPK3 mutant vs. WT parasites upon ionophore treatment. Although TgCDPK1 itself did not emerge from our stringently filtered datasets as differentially phosphorylated in the mutants vs. WT, these results indicate that there might be an increase in activity of TgCDPK1 in the TgCDPK3 mutants. Hence, we specifically looked for evidence of differences in the phosphorylation state of TgCDPK1 in our datasets and found several phosphopeptides with high-quality quantifications in the “EC/ION^+^_FW” condition that corresponds to the activation loop threonine (T200) of TgCDPK1 [39], [40]. The phosphorylation site identified in TgCDPK1 showed a higher level of phosphorylation (2.9-fold) in the mutant vs. WT parasites (Figure 4c), while the protein levels of TgCDPK1 appear similar between WT and TgCDPK3 mutants in our proteomic dataset (Supplemental Table S1) and by Western blot (data not shown). This phosphorylation is a prerequisite for activity of the kinase and supports the model that the TgCDPK3 mutants have a higher level of activated TgCDPK1. It was identified in no other dataset which is why it is not included in the set of 156 phosphorylation sites discussed above (all of which were seen in at least two datasets), but all peptides containing quantitative information for TgCDPK1:T200 were identified in the heavy and the light version in two different fractions giving confidence in its identification and quantification. All phosphopeptides were identified as a missed cleavage form, which is often the case for phosphopeptides [41]. We did not identify the activation-loop phosphosite in the dataset from the complemented mutants and so could not assess whether such complementation rescued its phosphorylation; however, we did observe hyperphosphorylation of the above-mentioned targets of TgCDPK1 in the MBE1.1 mutant and all showed phenotypic rescue upon TgCDPK3 complementation. Hence, TgCDPK3 plays a role in the phosphorylation of these proteins through another kinase, presumably, TgCDPK1.

### TgCDPK1 can partially rescue the egress phenotype of TgCDPK3

**Figure 5:**
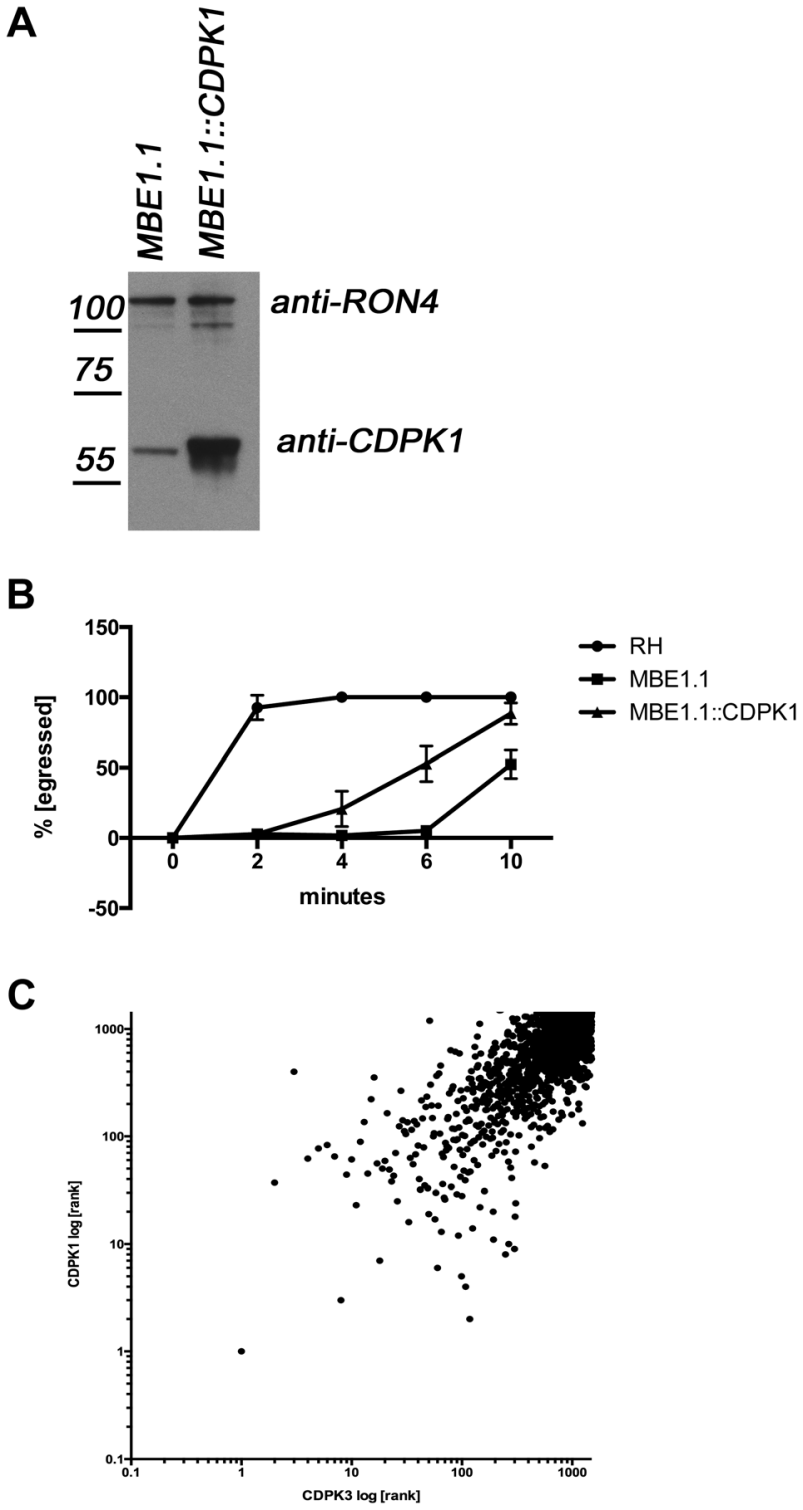
Overexpression of TgCDPK1 partially rescues the egress defect. **A**) TgCDPK1 expression in engineered parasites. Lysates from MBE1.1 parasites with and without TgCDPK1-HA expressed off the GRA2 promoter were separated by gel electrophoresis and blotted. Antibodies against TgCDPK1 were used to detect both the endogenous TgCDPK1 and TgCDPK1-HA expression (the endogenous protein migrates slightly faster than the HA-tagged version). The mass (in kDa) and migration of size markers are shown to the left RON4 expression was detected by blotting with rabbit anti-RON4 and served as a loading control. **B**) Overexpression of TgCDPK1 partially rescues egress. Parasites were treated with 1 µM A23187 and egress monitored over 10 minutes. Wild type (RH), TgCDPK3 mutant (MBE1.1) and MBE1.1 over-expressing TgCDPK1 (MBE1.1::CDPK1) were compared. **C**) TgCDPK1 and TgCDPK3 have overlapping substrate preferences. Peptide microarrays spotted with random 13-mer peptides with a central serine residue have been incubated with recombinant TgCDPK1 or TgCDPK1 and ^32^P-ATP. All peptides where ranked according to the ability of the respective kinase to phosphorylate any given peptide, with the highest phosphorylation ranked 1^st^. The scale is log10.

TgCDPK3 mutants egress in the presence of ionophore with much slower kinetics than WT parasites [8], [13]: whereas after 2 minutes 100% of WT parasites have exited in the presence of ionophore, TgCDPK3 mutants only start to slowly egress after 3–4 minutes, reaching near 100% egress levels by 10 minutes [13]. The increased amount of phosphorylated TgCDPK1 and its targets in the TgCDPK3 mutants prompted us to test whether accumulation of active TgCDPK1 might eventually reach levels necessary for this enzyme to take the place of TgCDPK3, thereby explaining the ability of the TgCDPK3 mutant parasites to respond, albeit slowly, to the ionophore. To test this hypothesis, we over-expressed TgCDPK1 in MBE1.1 parasites using a strong promoter (from the GRA2 gene) and measured the ability of MBE1.1::TgCDPK1 parasites to egress in the presence of ionophore. We tested the level of overexpression using an antibody specific to TgCDPK1, which recognizes a single band at around 55kD in MBE1.1 parasites (Figure 5A). The product of the introduced TgCDPK1 transgene is HA-tagged and showed the expected size-shift on gel electrophoresis with an expression level that was ∼10× higher than the slower migrating, endogenous TgCDPK1 (Figure 5A). Immunofluorescence imaging of TgCDPK1::HA showed a concentration of TgCDPK1::HA toward the periphery of MBE1.1::TgCDPK1 parasites whereas WT parasites showed a more general cytosolic staining; biochemical fractionation assays, however, revealed no difference in membrane association of TgCDPK1 in the two strains (data not shown). While we saw no difference in growth or number of parasites/vacuole in the MBE1.1::TgCDPK1 parasites (data not shown), they were substantially but not fully rescued in their ionophore-induced egress phenotype (Figure 5B): at 2 minutes, most WT (RH) parasites had egressed but MBE1.1 and MBE1.1::TgCDPK1 remained mainly inside; however, at 6 minutes ∼50% of MBE1.1::TgCDPK1 had egressed while MBE1.1 were still almost entirely intracellular. This shows that over-expression of TgCDPK1 can partially overcome the block seen in mutants lacking active TgCDPK3, supporting the implication of the SILAC data that the function of these two kinases is directly or indirectly linked.

### TgCDPK1 and TgCDPK3 have partially overlapping substrate specificity

We have previously shown that the egress phenotype of TgCDPK3 mutants can be rescued by overexpressing an engineered form of TgCDPK3 that has mutations in its predicted myristoylation and pamitoylation sites but only when using a strong promoter (SAG1), not when using the endogenous promoter [8]. This was likely because overexpressing TgCDPK3 allows a fraction of it to reach the plasma membrane [8] where it can phosphorylate its targets. To test whether the increased levels of activated TgCDPK1 observed in MBE1.1 mutants might similarly be rescuing the egress delay observed by phosphorylating TgCDPK3 targets, we directly compared the TgCDPK1 and TgCDPK3 substrate specificity. We incubated peptide microarrays spotted with ∼500 defined but random 13-mer peptides containing a central serine residue with recombinant TgCDPK1 or recombinant TgCDPK3 and compared their ability to phosphorylate the arrayed peptides (Figure 5C). We ranked the peptides according to the phosphorylation intensity with the highest phosphorylated peptide being 1^st^. A comparison of the ranking for each given peptide incubated with either CDPK1 or CDPK3 shows that a majority of the peptides are equally well phosphorylated by the two kinases although some are predominantly phosphorylated by one, but not the other. We did not obtain significantly enriched amino acids in any position for either TgCDPK1 or TgCDPK3, but that might be related to the technical limitation of these arrays. They sometimes contain more than 1 phosphorylatable residue per peptide; i.e., an additional serine, threonine or tyrosine residue in addition to the central serine. Since we cannot distinguish whether the central serine, or another phosphorylatable residue is phosphorylated, they have limited value for a motif analysis. However, it still allowed us to directly compare TgCDPK1 and TgCDPK3 in their linear motif analysis with the result that TgCDPK1 appears likely able to phosphorylate at least a subset of TgCDPK3 targets.

### Differential phosphorylation of calcium-dependent proteins in the absence of ionophore predicts a deregulation of calcium signaling and reveals elevation of basal calcium-levels in parasite lines with inactivated TgCDPK3

**Figure 6:**
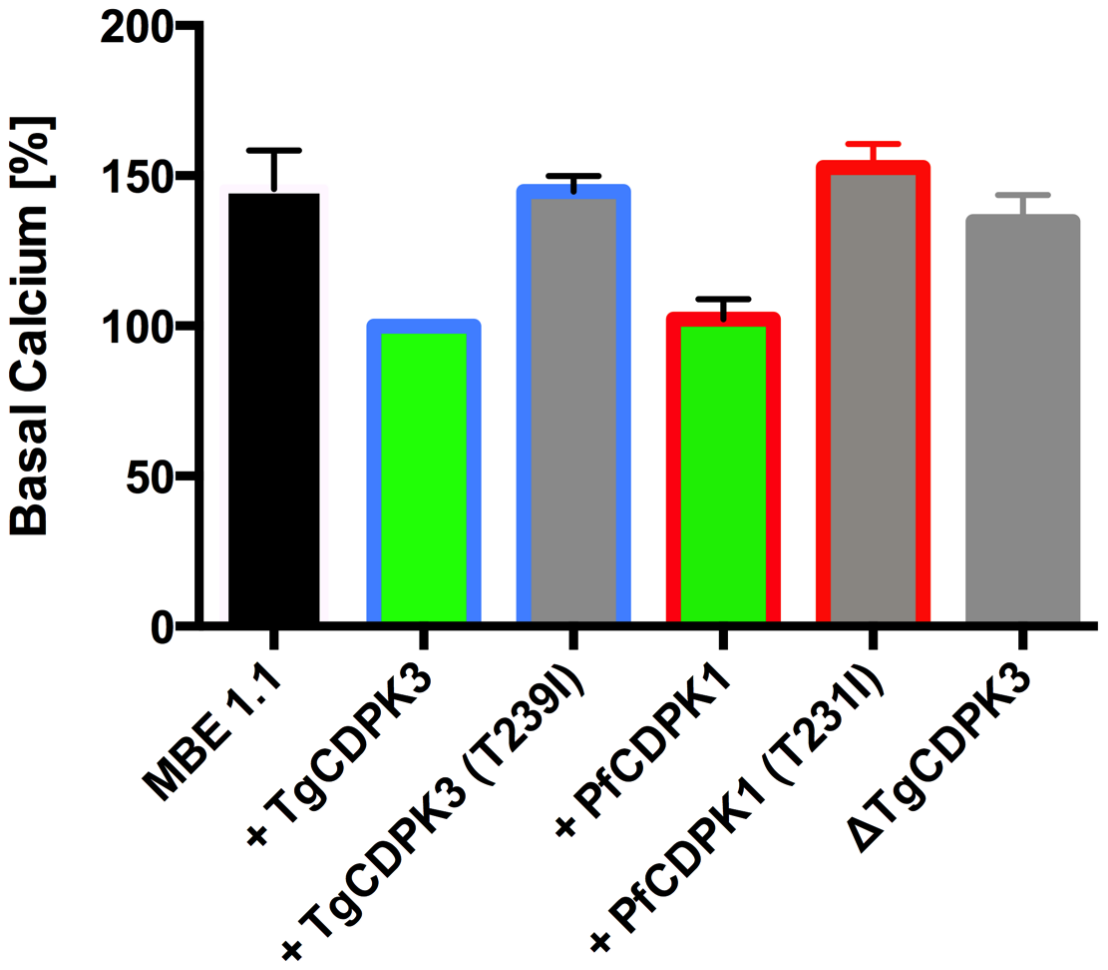
Absence of TgCDPK3 results in elevation of resting Ca^2+^ levels in *T. gondii* tachyzoites. Ca^2+^ levels in *Tgcdpk3* mutants (MBE 1.1), *Tgcdpk3* mutants complemented with either wild-type or kinase mutant versions of TgCDPK3 (T239I), ΔTgCDPK3 and *Tgcdpk3* mutants complemented with either wild-type or kinase mutant versions (T231I) of PfCDPK1 parasites was measured using the cell permeant calcium indicator Fluo-4-AM. Cell lines defective in egress are shown in grey, cell lines complemented with either WT TgCDPK3 or PfCDPK1 are capable of egress and are shown in green. Blue outlines show MBE1.1 complemented with TgCDPK3 variants while red outlines show data from MBE1.1 complemented with PfCDPK1 variants. Fluorescence values of MBE 1.1+ TgCDPK3 were set at 100% for relative comparison. *n* = 3 independent experiments. Error bars, SEM (^**^ P<0.01, one-way ANOVA). ns =  not significant.

In addition to the calcium-dependent proteins mentioned earlier, we identified a putative calcium-transporting ATPase (TGGT1_103910) that showed differential phosphorylation between mutant and WT parasites in the “IC/ION^−”^ condition. These results suggested a putative role for TgCDPK3 in regulating calcium levels. To test this, we measured calcium levels in the absence of ionophore in WT, MBE1.1, MBE1.1 complemented with a functional copy of TgCDPK3 (MBE1.1::CDPK3) and, as a control, MBE1.1 complemented with a nonfunctional copy of TgCDPK3 (MBE1.1::CDPK3(T239I)). Both, MBE1.1 and MBE1.1::CDPK3(T239I) showed elevated calcium levels (∼150%) compared to MBE1.1 complemented with a functional copy of CDPK3 (100%), supporting the notion that TgCDPK3 plays a role in maintaining normal intracellular calcium levels (Figure 6). To rule out that integration of the TgCDPK3 WT copy into MBE1.1 lowered basal calcium levels because of an off- target effect, we also measured basal calcium levels in MBE1.1 parasites complemented with the *Plasmodium falciparum* orthologue of TgCDPK3, PfCDPK1 (PF3D7_0217500), that has recently been shown to complement the egress phenotype of MBE1.1 [42]. Complementation with the WT PfCDPK1 version, but not a kinase dead mutant (T231I) decreases calcium levels similar to those of MBE1.1::CDPK3. No significant differences in the change of calcium levels were observed in the response to the calcium ionophore itself (data not shown). This suggests that inactivation of CDPK3 causes a reproducible elevation of basal calcium levels that are directly dependent on TgCDPK3.

## Discussion

The aim of this study was to identify phosphorylation events that are dependent on TgCDPK3. Based on previous publications, we hypothesized that the signaling pathways controlled by TgCDPK3 might be most easily detected during ionophore-induced egress. Among the proteins that fulfilled this prediction were several that are known to be secreted into the parasitophorous vacuole (PV), the PV-membrane (PVM) or into the host cell. Some of the phosphopeptides in these proteins showed a decreased abundance in the WT samples which could be due to dephosphorylation or degradation of these proteins resulting from breakdown of the PVM. We have not further investigated these events here, but they are consistent with the fact that breakdown of the PVM, normally an early event in egress, is defective in the TgCDPK3 mutant parasites [6]–[8], [13].

Despite the low number of phosphorylation sites for which we obtained SILAC ratios in untreated samples as discussed above, we identified several significant differential phosphorylation events on proteins that are either known or predicted to play a role in egress or associated processes, and these are discussed further below. In addition to sites that are differentially phosphorylated, those that show no change in phosphorylation status in the mutants relative to WT can be equally informative. For example, our results indicate that TgCDPK3 is not the major regulator of key phosphorylation sites observed on the components of the machinery that drives parasite motility including GAP45, one of the key “glideosome” proteins known to be regulated by phosphorylation [28], [30]. The only known part of the motor for which we confidently saw differences in the phosphorylation state between WT and TgCDPK3 mutants was MyoA. The differences observed for MyoA could explain some of the observed phenotypic differences in TgCDPK3 mutants with regards to motility, but given that at least one site (S20/S21) was already differentially phosphorylated in intracellular parasites not treated with ionophore (“IC/IONO^−^”), it appears that this phosphorylation site has a function independent of, or in addition to, egress. A recent report on a conditional knock-out of MyoA, where loss of this protein has no effect on parasite egress (or invasion [43]) supports the notion that phosphorylation of MyoA and other proteins by TgCDPK3 can serve functions other than egress. Interestingly, two other myosin isoforms (MyoG and MyoF) show up as differentially phosphorylated in the TgCDPK3 mutants vs. WT, one of which, MyoF, was recently described as playing a role in apicoplast segregation [44]. The role that these myosin isoforms play during ionophore-induced egress requires further analysis.

One protein strongly indicated as a potential regulator of motility is CAP, a regulator of actin dynamics [45], which we found to be ∼2-fold less phosphorylated in the TgCDPK3 mutants relative to WT in the presence of ionophore. In tachyzoites, CAP has been shown to be localized in the apical end, rapidly redistributing into the cytosol when becoming extracellular [46]. Thus, CAP could regulate actin dynamics during egress and motility in a location-dependent manner. In *Plasmodium berghei*, deletion of CAP showed a defect in oocyst development but the function of CAP and actin regulation in this process is not understood [23].

The identification of TgCDPK3-dependent phosphorylation of calcium-regulated proteins, including TgCDPK1, TgCDPK2a, and an EF-hand containing protein is a strong indicator that TgCDPK3, in addition to being regulated by calcium, controls other calcium-regulated processes. Furthermore, the identification of two proteins that are known phosphorylation targets of TgCDPK1, and indications that loss of TgCDPK3 may result in a more active TgCDPK1 itself, indicate that the pathways controlled by TgCDPK1 are, at least in part, activated in TgCDPK3 mutants treated with ionophore. While this suggests that TgCDPK3 might be a negative regulator of TgCDPK1, we have no direct evidence for this and understanding their precise roles will require further investigation.

Overexpression of TgCDPK1 partially rescues the egress phenotype. This indicates that 1) either active TgCDPK1 can phosphorylate targets of TgCDPK3, or 2) that active TgCDPK1 can activate egress and microneme secretion independent of TgCDPK3, but with much slower kinetics. Both kinases appear to have overlapping substrate specificity and the dominant localization of the overexpressed TgCDPK1 at the periphery and the increased rescue of egress supports the first option where TgCDPK1 may be phosphorylating TgCDPK3 targets at the plasma membrane. However, we cannot exclude the alternative, in which egress in the TgCDPK3 mutants is facilitated via a TgCDPK3-independent pathway. In both scenarios, the elevated levels of TgCDPK1 we observed in MBE1.1 parasites that are phosphorylated in the autophosphorylation loop might explain how TgCDPK3 mutant parasites egress slowly over time when treated with ionophore.

A possible explanation for how CDPK1 might be activated is via a PKG (TGGT1_087710) controlled pathway. Lourido *et al*., have shown that activation of PKG is partially CDPK3 dependent as inhibition of CDPK3 decreases egress triggered by Zaprinast [6]. PKG has also been shown to be important for egress in *Plasmodium falciparum* as a key-regulator for calcium levels thought to be important for regulation of the TgCDPK1 orthologue in *P. berghei*, PbCDPK3 [47], [48]. While we consistently identified the phosphorylated activation loop of TgPKG (Threonine 837, Supplementary table S1) under all conditions, we did not observe CDPK3- dependent differences. But it is possible that other phosphorylation sites of the protein, which we might not have detected for technical reasons, or other regulatory mechanisms are mainly involved in regulation of PKG.

Whatever the mechanism by which TgCDPK3 and PKG are connected, our data support a broader role of TgCDPK3 and the pathways it controls. This is evident from the proteins that are differentially phosphorylated, including proteins that are important for metabolism, transcription and ion homeostasis in addition to the proteins important for egress, several of which are differentially phosphorylated even in the absence of ionophore. This is in line with our observation that basal calcium levels are elevated in TgCDPK3 mutants in the absence of ionophore. This allows for a model in which altered calcium fluxes in TgCDPK3 mutants have a profound effect on the homeostasis of the cell, which could dictate how a cell behaves under conditions of stress. The fact that CDPK3 mutant parasites don't show a measurable phenotype in cell culture points towards compensatory mechanisms that allow for normal growth. The fine balance the parasites have to strike might be easily tipped as in the case of calcium ionophore treatment, when the egress phenotype becomes evident. While this phenotype was only observed in vitro, CDPK3 mutant parasites also have a phenotype *in vivo*.

Type I *Toxoplasma* parasite strains lacking TgCDPK3 (as used in this study) are still highly virulent in mice whereas Type II strains lacking TgCDPK3 activity are attenuated with severely reduced latent stages (tissue cysts) being found in the brain of chronically infected animals [8], [49]. We previously hypothesized that this could be due to an egress phenotype but the data we present here would suggest that the phenotypic changes are due to alterations in TgCDPK3-dependent functions unrelated to egress.

Our observation that there are differences in protein abundance in the TgCDPK3 mutants relative to WT resembles a recent study of the closest orthologue of TgCDPK3 in the malaria parasite *Plasmodium berghei*, PbCDPK1, that showed a role for PbCDPK1 in translational repression [10]. However, our results differ in two ways: 1) we observe differences in the absence of environmental triggers and 2) complementation of the TgCDPK3 mutants with a WT copy of the gene restores protein levels in only one case. The fact that we identified a substantial fraction of non-complemented phosphorylation sites in both the TgCDPK3 point mutant (MBE1.1) and knock-out clearly indicates that the effect is due to TgCDPK3. Several of these sites can be explained by changes in protein level indicating that phosphorylation state and proteome changes occur in the absence of TgCDPK3 and their non-complementation suggests that an epigenetic event “locks in” some of the effects. While it is possible that genetic effects in the chemical mutants are partially responsible for the observed static changes of protein levels, an epigenetic effect seems more likely as the only protein coding mutation identified in MBE1.1 using whole genome sequencing was in TgCDPK3. In addition to that, several of the changes that do not revert are identified in the chemical mutant and the TgCDPK3 knock-out strain, strongly arguing for a TgCDPK3 specific effect. Among the proteins and phosphorylation sites that are not complemented are some that are important regulators in glycolysis and other metabolic pathways, suggesting loss of TgCDPK3 could lead to long-lasting phenotypic changes that will not be reversed upon complementation. The results presented here, therefore, present a new insight into how the protein kinases of *Toxoplasma* interact to regulate several key functions, extending well beyond ionophore-induced egress.

## Materials and Methods

### Parasite culture and SILAC labeling

Parasites lines and labeling of parasites was achieved as previously described [15], [16]. Briefly: parasites were grown in heavy (146 mg/l ^13^C_6_-, ^15^N_2_-L-lysine, 84 mg/l ^13^C_6_-L-arginine, 40 mg/L unlabeled L-Proline) or light media (146 mg/l unlabeled L-lysine, 84 mg/l unlabeled L-arginine, 40 mg/l unlabeled L-Proline). After 4 complete lytic cycles, parasites incorporated between 96% and 98% of heavy amino acids. For the comparative analysis parasites were seeded onto confluent human foreskin fibroblast in 150 mm dishes with an MOI of 5 in either heavy or light media. 24 hours post-infection the cells were washed once with fresh media and incubated in the presence of 1 uM A23187 or DMSO for 30 seconds. After the incubation time, the parasites were immediately placed on wet ice and quickly washed once with pre-chilled, ice-cold PBS prior to lysis in ice-cold 8 M urea containing protein and phosphatase inhibitors (Roche). We performed each experiment using 5 individual 15 cm dishes in order to monitor an average of the signaling events that take place during the first 30 seconds of ionophore treatment.

### Isolation of phosphopeptide and non-phosphorylated peptides

Peptide and phosphopeptide samples were prepared as previously described [17], [18] using SCX and IMAC chromatography for phosphopeptide enrichment. Briefly, samples were lysed, reduced, alkylated, and digested with trypsin. After desalting, the peptides were fractionated using strong cation exchange chromatography (SCX) and phosphopeptides were further enriched using IMAC (immobilized metal affinity chromatography) and the phosphorylated and non-phosphorylated flow-through peptides were analyzed by LC_MS/MS on a LTQ-Velos Orbitrap in technical duplicates in a total of 196 MS/MS runs. In addition, we analyzed the phosphoproteome and proteome of RH vs. the MBE1.1 mutant complemented with a WT copy of TgCDPK3 (MBE1.1::CDPK3; [8]) in technical duplicate in comprised of a total of 36 runs. This was done using parasites “EC/ION^+^”.

### Mass-spectrometry

Phosphorylated and non-phosphorylated (flow-through) peptides were resuspended in 4% formic acid, 5% acetonitrile and analyzed by LC-MS/MS in technical duplicate on a system consisting of a MicroAS autosampler (Thermo Scientific), binary HPLC pumps (Agilent 1200 series) with flow-splitting, an in-house built nanospray source, and an LTQ Orbitrap Velos (Thermo Scientific). 2 µg of sample was loaded onto a 100 µm ID fused silica capillary packed with 18 cm of 5 µm Magic C18AQ resin (Michrome Bioresources). Peptides were eluted using a gradient of water:acetonitrile with 0.1% formic acid from 7% to 25% acetonitrile over 120 min, and then 25–40% B over 30 minutes. A top 10 method was run consisting of one MS1 scan (resolution: 6×10^4^ AGC: 5×10^5^, maximum ion time: 500 ms) followed by ten data dependent MS2 scans (AGC: 1×10, maximum ion time: 100 ms) of the most abundant ions. Dependent scans were configured with the following settings: 2.0 m/z isolation width, dynamic exclusion width: −0.52, 2.02, exclusion duration: 60 seconds, normalized collision energy: 35, activation time: 5 ms. Charge state screening was employed to reject ions with unassigned or +1 charge states.

### Egress assays

HFFs were cultivated in 96 well plates and each well infected with 500 parasites. After 24 hours of growth, the parasites were incubated in HBSS containing 1 µM A23187 calcium ionophore for time periods ranging from 0 to 10 minutes, in triplicates, after which the cells were fixed in 100% methanol and subsequently stained with Giemsa. All assays have been done on at least three independent occasions.

### Data analysis

Spectra were searched against a concatenated database of Human (IPI, version 3.66) and *Toxoplasma* (toxoDB, release 6.1) proteins using SEQUEST [50], with 15 ppm precursor mass tolerance, trypsin specificity with up to two missed cleavages, static modification of cysteine (carbamidomethylation, +57.0215) and variable modification of serine, threonine, and tyrosine (phosphorylation, +79.9663), methionine (oxidation, +15.9949) lysine (SILAC 13C(6)15N(2), +8.0142), and arginine (SILAC 13C(6), +6.0201). Phosphorylation site localization was assessed using the Ascore algorithm [20]. All datasets were filtered using the target-decoy method [19], [51] to a false discovery rate (FDR) of <1% on the peptide and <3% on the protein level. Phosphopeptides were combined into phosphosites based on their localization probabilities, and phosphosites were further filtered to an FDR of <1% for each phosphorylatable residue (S,T,Y) using the peptide score provided by Ascore. All peptides matching to the human proteome were removed to exclude peptides from the parasite that are identical with the host and where quantification is thus not reliable. SILAC quantification was achieved by analysis of the MS1-intensity peaks using the VISTA algorithm [52]. Quantifications were scored using: closeness of log_2_ H/L to 1∶1, signal to noise of each isotopic partner, and a VISTA confidence metric that accounts for chromatography quality. Weighted averages were calculated using these scores for sites and proteins for which more than one identification was made. These weighted averages are intentionally conservative, in an attempt to eliminate false-positives from the tails of the distribution. Unweighted average and standard deviation calculations have been included as well. Phosphopeptides were categorized as either mono-, bis-, or tris-phosphorylated, and separate averages were calculated for sites found in peptides of each type. The resulting data was further filtered for a minimum of 2 quantifications in each respective experiment, a minimum VISTA confidence score of 88 for the best quantification and a minimum signal to noise ratio for the best peptide of 8.

SILAC log_2_ ratios were centered on “0” based on the median SILAC ratio for each dataset.

Differentially phosphorylated sites were identified by using the following criteria:

a minimum log_2_ fold change of 0.75 (+ or −), which is ∼1.5 times the standard deviation across the experimental datasets, and a consistent change of phosphorylation site abundance in one or more of the conditions. Note, one mismatch was allowed to capture phosphorylation sites that are just at the threshold, or missing in a single sample due to a bad SILAC quantification, to be included in the dataset. Phosphorylation site quantifications were manually curated by analyzing the MS1 elution profiles and removed if they originated from low-quality quantifications.

Protein SILAC ratios were calculated by using the median SILAC ratio for all identified peptides. Pearson correlation was calculated using a two-tailed test with a 95% confidence interval (Prism6).

### Generation of TgCDPK1 overexpressing parasites

The open reading frame of TgCDPK1 was cloned into pGRA [53] using the restriction sites NsiI and NcoI. The plasmid was linearized using HindIII and transfected into MBE1.1 parasites and selected with MPA/XAN as previously described [53].

### Peptide-microarray analysis

Random peptide library kinase arrays (KIN-MA-RLYS, JPT, Germany) with a central serine were incubated according to the manufacturer's instructions. Briefly, 100 nM recombinant CDPK1 or CDPK3 was incubated with 50 µM Ca and 10 µM cold ATP to allow for autophosphorylation and activation of the kinase. This mix (400 µL) was then supplemented with 10 µCi of ^32^P-ATP and added to the peptide arrays for 4 h at 30 degrees Celsius. After washing and drying a high-resolution phosphor-imaging screen (Fujifilm, BAS-SR) was exposed. Images were acquired using a Typhoon scanner using 25 µM resolution and spot intensity values were analyzed using microarray software. Median spot intensities were generated after manual verification of each spot. Contaminations were manually removed and excluded from the analysis.

Each median intensity was plotted as a rank from highest (1^st^ rank) to the lowest observed phosphorylation.

### Calcium measurements assay

Naturally egressed parasites were resuspended in intracellular buffer (5 mM NaCl, 142 mM KCl, 2 mM EGTA, 1 mM MgCl_2_, 5.6 mM glucose, 25 mM HEPES, pH 7.2) and labeled with Fluo-4 AM (2.5 µM) at room temperature with shaking for 30 minutes. Parasites were then pelleted, washed and resuspended in intracellular buffer followed by incubation at room temperature with shaking for 30 minutes to allow de-esterification. The parasites were then loaded onto 96-well plates (10^7^ per well) and fluorescence (excitation 496 nm and emission 516 nm) was quantified using a Synergy H1 plate reader (BioTek).

### Antibodies

Recombinant full length TgCDPK1-HIS6 was generated by cloning the open reading frame into the expression vector pET28. Recombinant TgCDPK1 was purified using the HIS-tag with NI-NTA agarose (GE-Healthcare). 200 µg of purified TgCDPK1 was injected with an equal volume of Freunds incomplete adjuvant into 6–8 week old pre-screened BALB/c mice from Charles Rivers. None of the mice showed reactivity in the pre-bleeds. Mice were boosted 3 times with 100 µg of recombinant protein every 3 weeks. Test bleeds were taken at week 6 and all mice were sacrificed at week 10 for the terminal bleed. Final bleed serum was screened for selectivity by Western blot and used in this study.

### Software used in this study

Statistical analysis and graphs were made using GraphPad Prism6. Data handling was performed using Excel and MySQL using Python scripts. Heat-maps were generated using Cluster 3 (unclustered, k-means), and visualized by Treeview.

## Supporting Information

Figure S1 Experimental conditions used in this study.
(TIF)

Figure S2 Heatmap of all phosphorylation sites that differ between WT and TgCDPK3 mutant parasites, but where the protein-level is unchanged. The color bar represents log_2_ SILAC ratios.
(TIF)

Table S1 All quantified phosphorylation sites and proteins identified in this study. All quantified mono-, bis- and tris-phosphorylated peptides that passed the filtering are shown on individual sheet. Additional information from the individual mass-spectrometry experiments are represented in individual sheets.
(XLSX)

Table S2 A list of all phosphorylation sites that show differential phosphorylation site usage between CDPK3 mutants and wild type parasites.
(XLSX)
