# Supplementary figures and images for: The Calcium-Dependent Protein Kinase 3 of Toxoplasma Influences Basal Calcium Levels and Functions beyond Egress as Revealed by Quantitative Phosphoproteome Analysis
Source: PLoS Pathog. 2014 Jun 19;10(6):e1004197. doi: 10.1371/journal.ppat.1004197 (PMC4063958; doi:10.1371/journal.ppat.1004197)

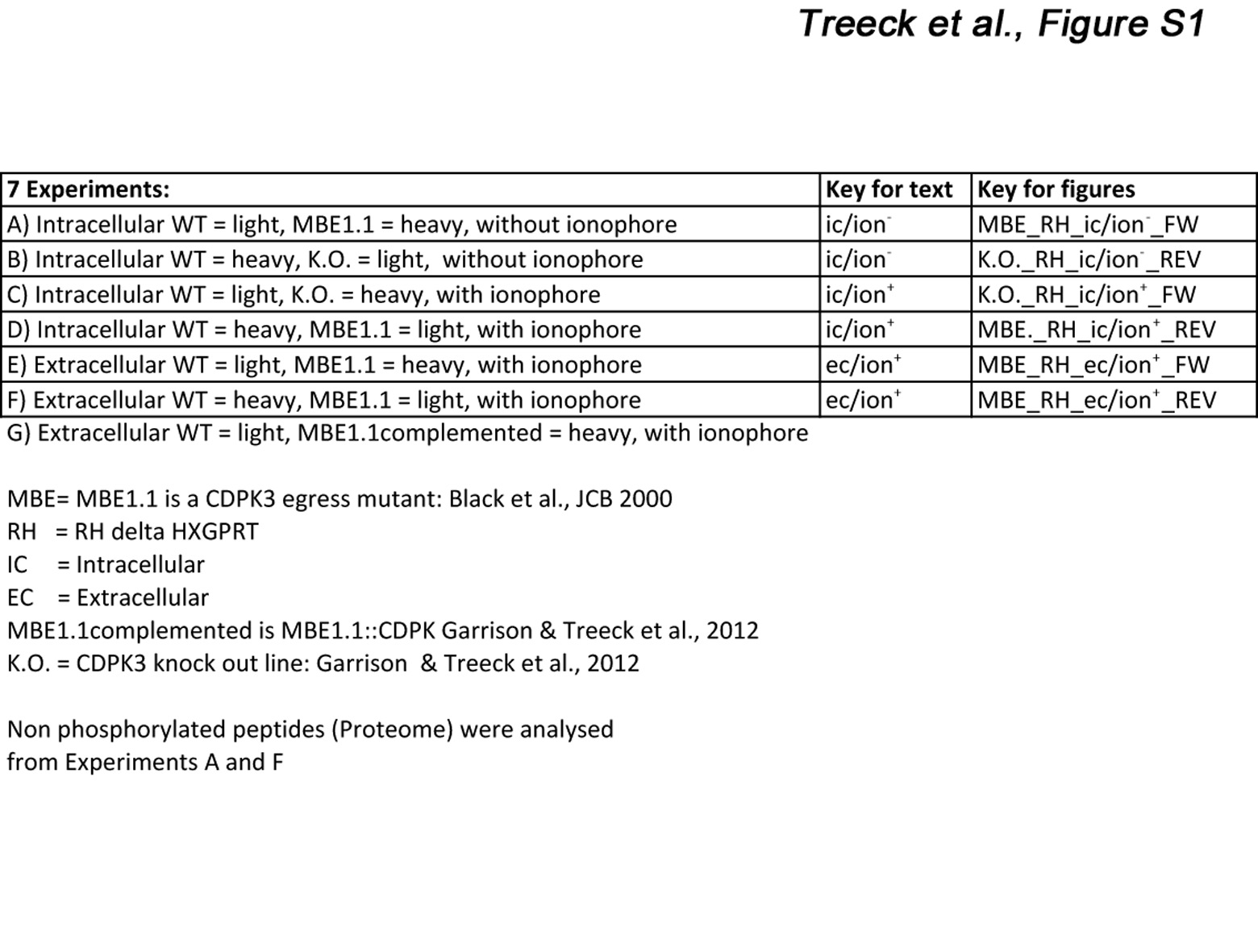

Supplement: Figure S1 — Experimental conditions used in this study. (TIF) [file ppat.1004197.s001.tif]

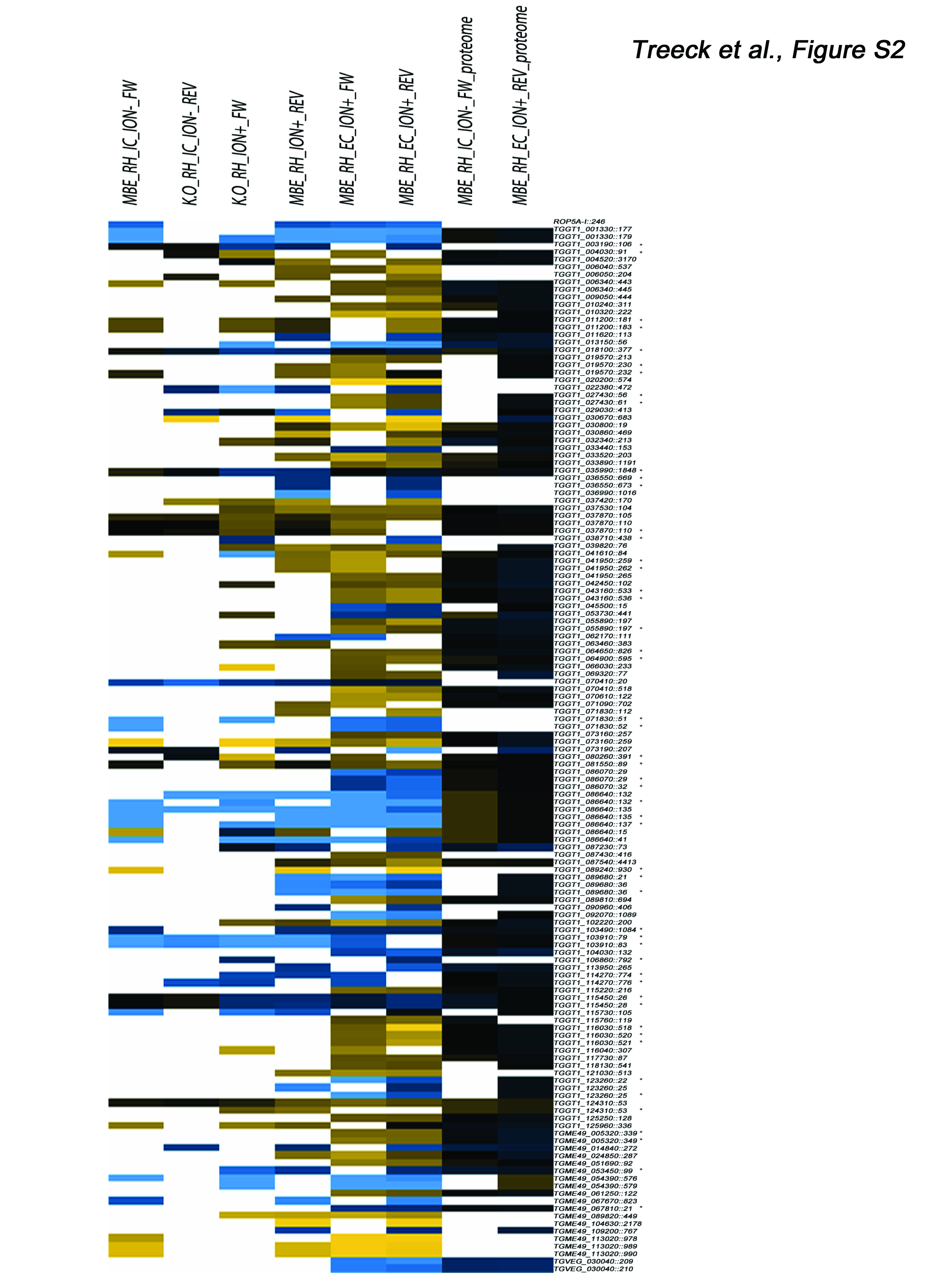

Supplement: Figure S2 — Heatmap of all phosphorylation sites that differ between WT and TgCDPK3 mutant parasites, but where the protein-level is unchanged. The color bar represents log2 SILAC ratios. (TIF) [file ppat.1004197.s002.tif]
